# LncRNA 148400 Promotes the Apoptosis of Renal Tubular Epithelial Cells in Ischemic AKI by Targeting the miR−10b−3p/GRK4 Axis

**DOI:** 10.3390/cells11243986

**Published:** 2022-12-09

**Authors:** Xingjin Li, Zhifen Wu, Jurong Yang, Dongshan Zhang

**Affiliations:** 1 Department of Emergency Medicine, Second Xiangya Hospital, Central South University, Changsha 410011, China; 2 Emergency Medicine and Difficult Diseases Institute, Second Xiangya Hospital, Central South University, Changsha 410011, China; 3 Department of Nephrology, Second Xiangya Hospital, Central South University, Changsha 410011, China; 4 Department of Nephrology, The Third Affiliated Hospital of Chongqing Medical University, Chongqing 401120, China

**Keywords:** AKI, lncRNA148400, miR−10b−3p, GRK4, apoptosis

## Abstract

Although recent studies have reported that long non-coding RNA (lncRNA) is involved in the development of ischemic acute kidney injury (AKI), the exact function and regulatory mechanism of lncRNAs in ischemic AKI remain largely unknown. Herein, we found that ischemic injury promoted the expression of lncRNA 148400 in mouse proximal tubule-derived cell line (BUMPT) and C57BL/6J mice. Furthermore, the lncRNA148400 mediates ischemic injury-induced apoptosis of BUMPT cells. Mechanistically, lncRNA 148400 sponged miR−10b−3p to promote apoptosis via GRK4 upregulation. Finally, knockdown of lncRNA 148400 alleviated the I/R-induced deterioration of renal function, renal tubular injury, and cell apoptosis. In addition, cleaved caspase−3 is increased via targeting the miR−10b−3p/GRK4 axis. Collectively, these results showed that lncRNA 148400/miR−10b−3p/GRK4 axis mediated the development of ischemic AKI.

## 1. Introduction

Acute kidney injury (AKI) is a common and severe disease, characterized by a significant increase in serum creatinine level over a short time period and is associated with a sudden decrease in urine volume [1,2]. Patients with severe AKI have a high mortality rate of almost 40–60% [1,3]. Ischemia-reperfusion injury (I/R) is one of the most common causes of AKI [4]. To date, there is no effective method to prevent AKI progression, except for dialysis and renal transplant. Hence, we explored the underlying pathogenesis of AKI.

Long non-coding RNAs (lncRNAs) have a length of more than 200 nucleotides [5]. Several studies have reported that unusual expression of lncRNAs is significantly associated with multiple diseases, such as cancer [6], liver [7], heart [8], and kidney diseases [9]. Recent studies have also reported that lncRNAs are closely related to AKI progression. For example, lncRNAs Meg3, Malat1, GAS5, NEAT1, and LOC105374325 promoted the development of ischemic AKI [10,11,12,13,14]. By contrast, lncRNAs H19, TUG1, TCONS_00016406, and PRNCR1 protected against the progression of ischemic AKI [15,16,17,18]. In addition, several lncRNAs sponge microRNAs to adjust target gene expression [19,20]. The lncRNA ENSMUST00000 148400 (termed lncRNA148400), is located on chromosome 3 (chr3:93561387+93561505). However, the expression, function, and regulatory mechanism of lncRNA ENSMUST00000 in I/R-induced AKI remain unknown.

The present study is the first to report that lncRNA 148400 is induced by ischemic injury in vitro and in vivo. Functionally, lncRNA148400 promotes apoptosis of BUMPT cells during ischemic injury. Mechanistically, lncRNA 148400 targets miR−10b−3p to increase GRK4 expression. Finally, lncRNA 148400 siRNA alleviates I/R-induced AKI via regulation of the miR−10b−3p/GRK4 axis.

## 2. Materials and Methods

### 2.1. Antibodies and Reagents

The antibody GRK4 (Cat. No. 11808-1-AP) was obtained from Santa Cruz Biotechnology (Santa Cruz, CA, USA). Anti-cleaved caspase−3 (Cat. No. 9661s) was purchased from Cell Signaling Technology (Danvers, MA, USA). Anti-Caspase-3 (Cat. No. ab184787) was obtained from Abcam (Cambridge, MA, USA). Anti-β-Tubulin (Cat. No. T0023) was purchased from Affinity Biosciences (Cincinnati, OH, USA). The luciferase assay kit was obtained from BioVision (Milpitas, CA, USA). The lncRNA 148400 siRNA, miR−10b−3p mimic, miR−10b−3p inhibitor, GRK4 siRNA, and GRK4 plasmid were purchased from Ribo (Guangzhou, China). Lipofectamine 2000 was obtained from Life Technologies (Carlsbad, CA, USA). TRIzol reagent was purchased from Invitrogen (Carlsbad, CA, USA). The Ag SYBR Green Pro TaqHS premix was obtained from Accurate Biotechnology (China).

### 2.2. Cell Culture and Treatments

BUMPT cells were cultured in DMEM (Sigma-Aldrich, St. Louis, MO, USA) supplemented by 10% fetal bovine serum and antibiotics (100 U/mL penicillin G and 100 µg/mL streptomycin) at 37 °C in a humidified atmosphere of 5% CO_2_ and 95% air. When the cell density reached about 90%, ATP depletion cell model was established using antimycin A and calcium ionophore, as described previously [21,22]. Lipofectamine 2000 was used for transfection of lncRNA 148400 siRNA, miR−10b−3p mimic, miR−10b−3p inhibitor, GRK4 siRNA, GRK4 plasmid, and the negative control.

### 2.3. Ischemic AKI Model

Male C57BL/6J mice aged 8–10 weeks were purchased from Shanghai Animal Center (Shanghai, China) and maintained under a 12-h light/dark cycle with free access to food and water. Before the I/R injury model was established, the lncRNA 148400 siRNA (15 mg/kg per injection) was injected into the tail vein of male C57BL/6J mice twice a day for 1 day [23]. The renal blood supply was blocked for 28 min and then restored for 24 or 48 h [22,24].

### 2.4. RT−qPCR Analysis

The total RNA was extracted from BUMPT cells and mouse kidney cortex using trazil. Then, 2 μg of trazil was reversed to single-stranded DNA using Evo M-MLV. RT−qPCR was performed using LightCycler^®^ 480 II (Basel, Switzerland). Ag SYBR Green Pro TaqHS premix was used according to the manufacturer’s instructions. The sequences of lncRNA 148400 were retrieved from the Ensembl database (Gen ID: ENSMUST00000148400.2). The primer sequences were as follows: lncRNA 148400, 5′- AGACCACTTGACAAAGGAGGAC-3′ (forward) and 5′- ATAGAAGAAAG GGAAGGGCACTC-3′ (reverse); miR−10b−3p,5′-CGCGCAGATTCGATTCTAGG-3′ (forward) and 5′-AGTGCAGGGTCCGAGGTATT-3′ (reverse); GRK4,5′-CGAGGAAGAGTTGGTACTG TTGGC-3′ (forward) and 5′-ACAGCCAAGTCCCCACCAGTC-3′ (reverse); β-actin, 5′-GGCT GTATTCCCCTCCATCG-3′ (forward) and 5′-CCAGTTGGTAACAATGCCATGT-3′ (reverse); U6, 5′-CTCGCTTCGGCAGCACA-3′ (forward) and 5′-AACGCTTCACGAATTTGCGT-3′ (reverse). Quantification was performed using ΔΔCt values.

### 2.5. Immunoblot Analysis

Western blotting was performed as described previously [25,26,27,28]. Whole cell and renal cortex lysates were detected using SDS/PAGE and transferred to nitrocellulose membrane, blocked in 5% milk, incubated with the corresponding primary and secondary antibodies, and then detected using Western lightning-enhanced chemiluminescence reagent.

### 2.6. Fluorescence In Situ Hybridization (FISH) Analysis

Fluorescent probes of lncRNA 148400 and miR−10b−3p, U6, and 18S were purchased from Ribo. The nuclei of BUMP cells were stained with U6, the cytoplasm was stained with 18S, and lncRNA 148400 was labeled with Cy3. The sections from BUMPT cells and mouse kidneys were hybridized with the corresponding probes overnight and then stained with DAPI. Fluorescence imaging was performed using a laser scanning confocal microscope.

### 2.7. Flow Cytometry (FCM) Analysis of Apoptosis

Apoptosis was examined using annexin V-FITC/PI staining. The BUMPT cells were digested and collected using 0.25% trypsin without EDTA. The cells were washed twice with cold PBS. According to the manufacturer’s instructions for Annexin V apoptosis detection kit (Cat. No. 556547; BD Pharmingen, Franklin, NJ, USA), the cells were resuspended with binding buffer and incubated in the dark for 15 min after Annexin V staining. Then, the cells were stained with PI for 5 min. Finally, 200 μL of binding buffer was added to detect cell apoptosis.

### 2.8. Luciferase Reporter Assays

Luciferase reporter assays were conducted as described previously [29,30,31]. Dual-luciferase reporters of GRK4-3′UTR (WT-Luc-GRK4), GRK4 (MUT-Luc-GRK4), lncRNA 148400 (WT-Luc-lncRNA148400), or lncRNA 148400 (MUT-Luc-lncRNA148400) were constructed and co-transfected with amiR−10b−3p mimic or scrambled into BUMPT cells for 48 h. Then, luciferase activity was examined using SpectraMax M5 (Molecular Devices, Sunnyvale, CA, USA) and normalized to pGMLR-TK activity.

### 2.9. Renal Function and Morphology

The levels of serum creatinine and urea nitrogen were used to evaluate blood and renal function, respectively, according to the instructions of renal function examination kit (Nanjing Jiancheng Bioengineering Institute, Jiangsu, China). The morphology of renal tissue was assessed using hematoxylin and eosin (H&E) staining [32,33,34]. In addition, TUNEL staining was used to assess apoptosis [35,36,37].

### 2.10. Statistical Analysis

The two groups were compared using two-tailed Student’s *t*-tests. Multiple group comparisons were performed using one-way ANOVA or Two-way ANOVA. Quantitative data are expressed as mean ± SD. Differences with *p* < 0.05 were statistically significant.

## 3. Results

### 3.1. I/R-Induced Expression of lncRNA 148400 in BUMPT Cells and Mice Kidneys

**Figure 1:**
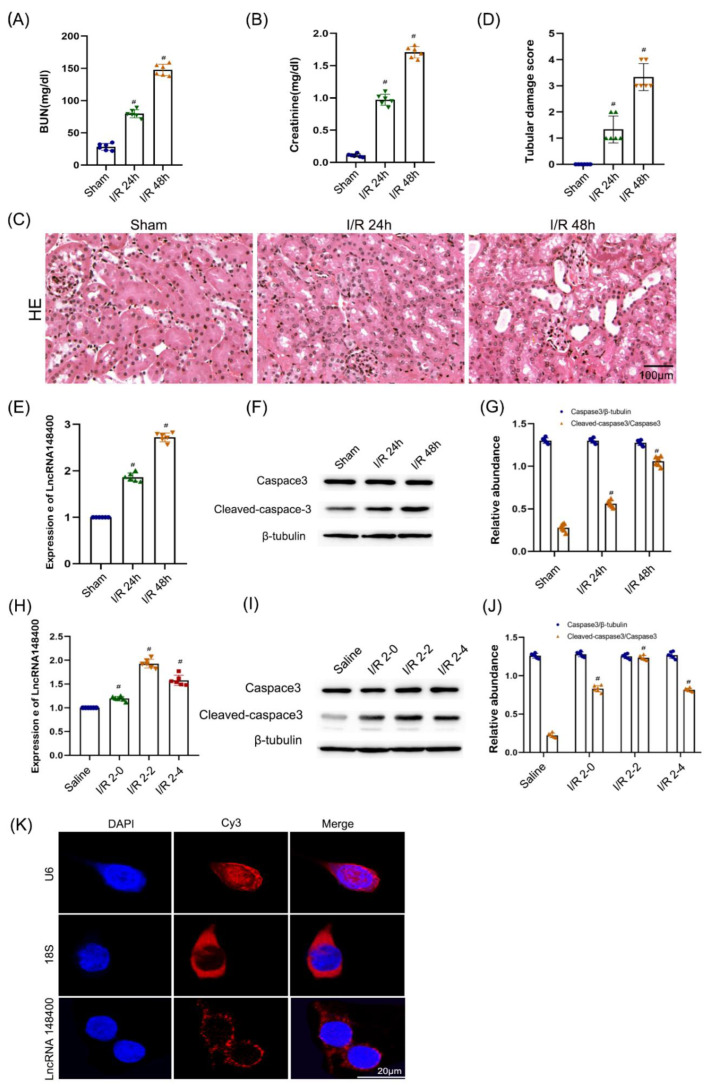
**I/R or ATP–depletion–induced the expression of LncRNA148400 in C57/BL6 mice and BUMPT cells**. C57/BL6 mice were subjected to the ischemic (I, 28 min)/reperfusion (R, 24 h or 48 h). BUMPT cells were treated with I (2 h)/R (0, 2, and 4 h). (**A**) BUN (**B**) Creatinine. (**C**) Representative HE staining. (**D**) Representative tubular damage score. (**E**) RT–qPCR analysis of the expression of lncRNA148400. (**F**) The immunoblot analysis of the expression of caspase 3 and cleaved caspase 3. (**G**) The gray analysis between them. (**H**) RT–qPCR analysis of the expression of lncRNA148400. (**I**) The immunoblot analysis of the expression of caspase3 and cleaved caspase3. (**J**) The gray analysis between them. (**K**) The FISH detection of intracellular localization of LncRNA 148400 in BUMPT cells. U6 was used as control of nucleus marker, while 18S was applied as control of cytoplasm marker. Score Bar is 20 µM. Data are expressed as mean ± SD (*n* = 6). ^#^ *p* < 0.05 vs. Sham group or Saline group.

We investigated whether lncRNA 148400 was involved in I/R-induced injury. Before this, we detected the renal function changes at I/R (28 min/24 h, 48 h and 72 h). The data showed that blood urea nitrogen (BUN) and serum creatinine concentrations were increased at 24 h after reperfusion, and then reached a peak at 48 h after reperfusion, and finally declined at 72 h after reperfusion (Supplementary Figure S1A,B). Hence, 48 h after reperfusion was selected as an observation point. First, C57/BL6 mice were subjected to I/R (28 min and 48 h). The serum levels of blood urea nitrogen (BUN) and creatinine were gradually increased at 24 h after reperfusion and reached a peak at 48 h after reperfusion (Figure 1A,B). Similarly, H&E staining indicated that I/R induced slight and moderate renal tubular injury at 24 and 48 h after reperfusion, respectively, which was further confirmed by the tubular damage score (Figure 1C,D). Furthermore, RT−qPCR analysis demonstrated that the mRNA level of lncRNA 148400 was gradually increased at the indicated time points (Figure 1E). The immunoblot analysis showed that the expression of cleaved caspase−3, but not caspase-3, was increased at 24 and 48 h after reperfusion (Figure 1F,G). Subsequently, RT−qPCR analysis indicated that the mRNA expression of lncRNA 148400 was upregulated at 0 h after oxygen deprivation by antimycin, attained a peak at 2 h afterward, and declined thereafter (Figure 1H). The trend of expression level of cleaved caspase−3 was consistent with that of lncRNA 148400 (Figure 1I,J). Finally, FISH analysis demonstrated that lncRNA 148400 was mainly localized in the cytoplasm of BUMPT cells (Figure 1K). These data suggest that the expression of lncRNA 148400 was increased under ischemic injury in vivo and in vitro.

### 3.2. LncRNA 148400 siRNA Ameliorates I/R-Induced Apoptosis of BUMPT Cells

**Figure 2:**
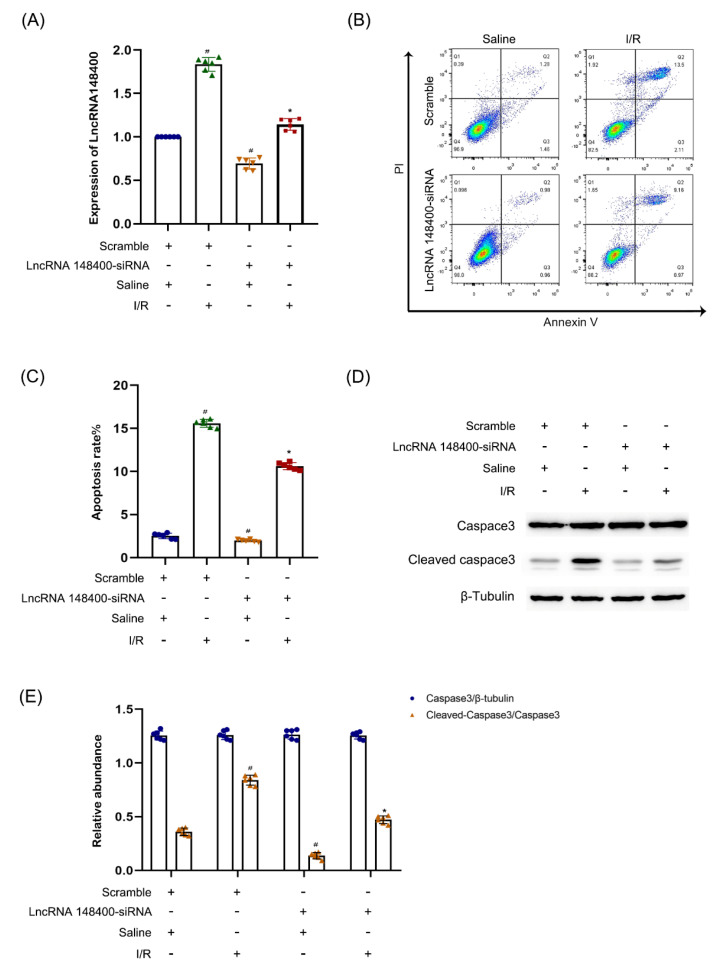
**LncRNA148400 siRNA attenuates I/R induced BUMPT cell apoptosis.** BUMPT cells were transfected with 100 nM lncRNA148400 siRNA or scramble, and then subjected to I(2 h)/R(2 h) injury. (**A**) RT−qPCR analysis of the expression of LncRNA 148400. (**B**) FCM analysis of apoptosis in BUMPT cells. (**C**) Representative apoptosis rate (%). (**D**) The immunoblot analysis of caspase 3 and cleaved-caspase3. (**E**) The gray analysis between them. Data are expressed as mean ± SD (*n* = 6). ^#^ *p* < 0.05 vs. Saline scramble group; * *p* < 0.05 vs. I/R with scramble group.

Next, we explored the function of lncRNA 148400 in ischemic injury. BUMPT cells were transfected with or without lncRNA 148400 siRNA and then treated with I/R (2 h each). The RT−qPCR showed that the expression level of lncRNA 148400 was markedly suppressed under basic and I/R treatment (Figure 2A). FCM results confirmed that lncRNA 148400 siRNA significantly suppressed I/R-induced cell apoptosis (Figure 2B,C). These results were similar to those of immunoblotting analysis for cleaved caspase−3 and caspase-3 (Figure 2D,E). These results indicated that lncRNA 148400 induces apoptosis in ischemic injury.

### 3.3. LncRNA 148400 Overexpression Enhances I/R-Induced Apoptosis in BUMPT Cells

**Figure 3:**
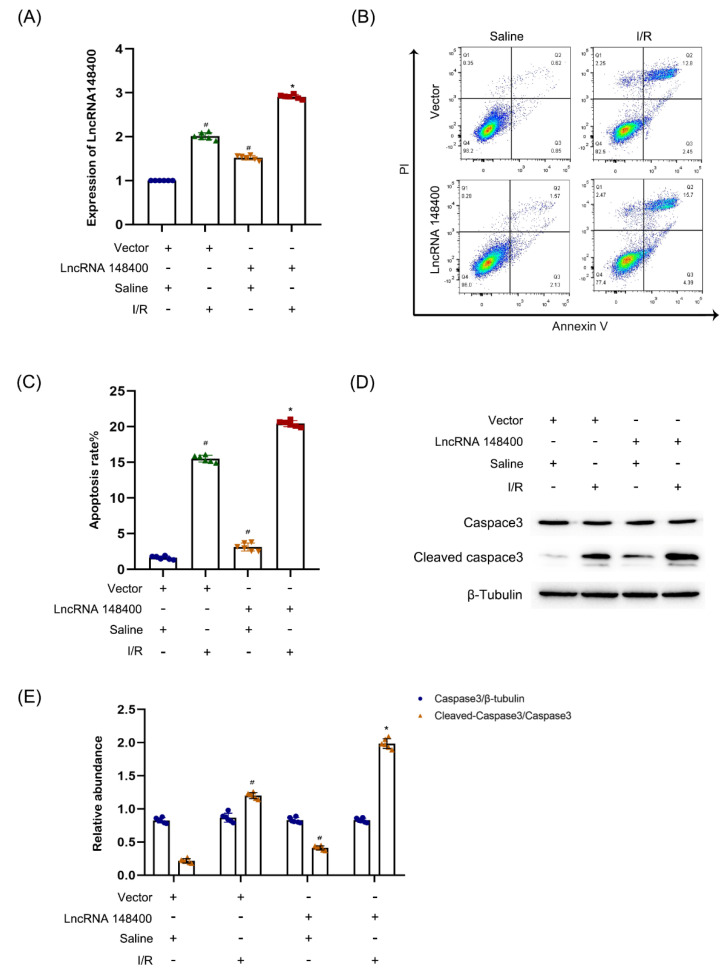
**Overexpression of LncRNA148400 enhanced I/R-induced apoptosis and increasing of Cleaved−caspase3 in BUMPT cells**. BUMPT cells were transfected with or without lncRNA148400 plasmid, and then treated with or without I (2 h)/R(2 h) injury. (**A**) RT−qPCR analysis of the expression of LncRNA 148400. (**B**) FCM analysis of apoptosis in BUMPT cells. (**C**) Representative apoptosis rate (%). (**D**) The immunoblot analysis of caspase 3 and cleaved−caspase 3. (**E**) The gray analysis between them. Data are expressed as mean ± SD (*n* = 6). # *p* < 0.05 vs. saline with scramble group; * *p* < 0.05 vs. I/R with scramble group.

Although we found that lncRNA 148400 knockdown attenuated the apoptosis caused by I/R, the effect of lncRNA 148400 overexpression on apoptosis remains unclear. The overexpression of lncRNA 148400 not only enhanced the lncRNA 148400mRNA level (Figure 3A) but also increased the apoptosis and expression of cleaved caspase−3 in BUMPT cells under standard and I/R conditions (Figure 3B–E). Our data confirmed that lncRNA 148400 had a pro-apoptotic effect in renal ischemic injury.

### 3.4. miR−10b−3p Was Sponged by the lncRNA 148400

**Figure 4:**
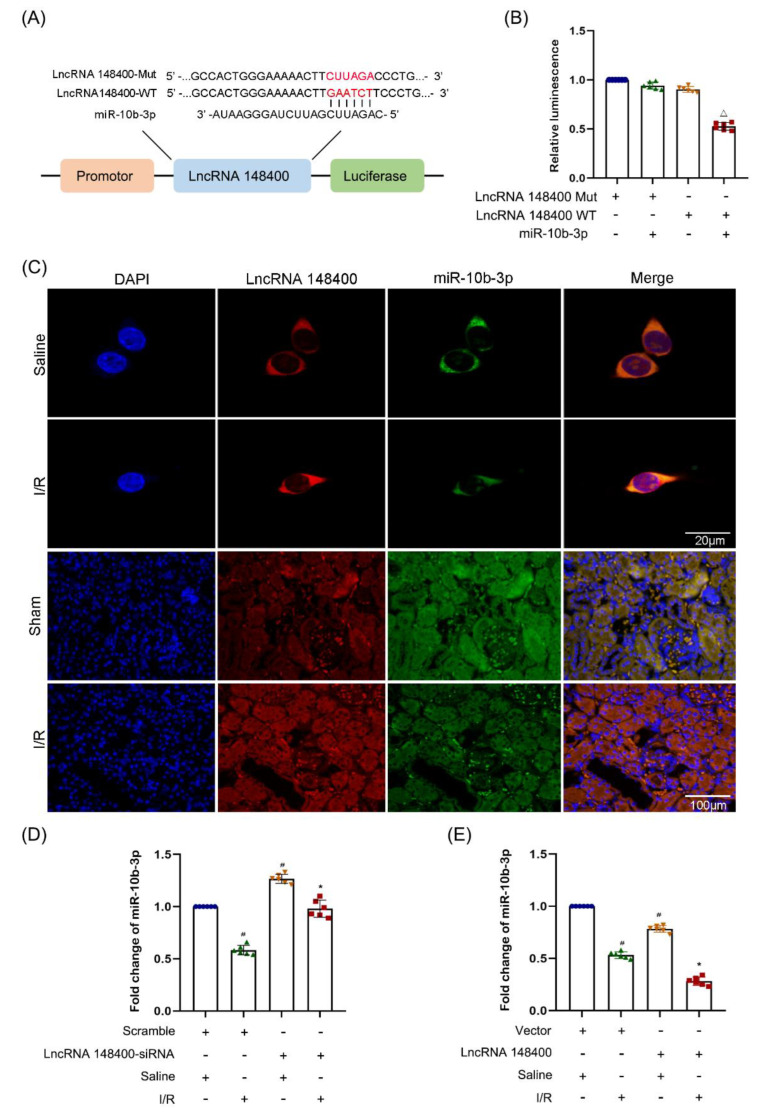
**LncRNA148400 sponged miRNA−10b−3p in vitro and vivo.** (**A**) Sequence alignment analysis of the complementary and mutated strand of LncRNA148400 and miRNA−10b−3p. (**B**) Detection of luciferase activities after co-transfection with lncRNA148400−WT or lncRNA148400−MUT plus with or without miRNA−10b−3p. (**C**) The FISH detection of intracellular co-localization of lncRNA148400 and miRNA−10b−3p in BUMPT cells and mice kidney under basic and I/R injury. (**D**,**E**) RT−qPCR analysis of the expression of miRNA−10b−3p. Data are expressed as mean ± SD (*n* = 6). Δ*p* < 0.05 vs. co-transfection of miR−10b−3p mimic and LncRNA148400 WT or LncRNA 148400−MUT groups. # *p* < 0.05 vs. saline with scramble group; * *p* < 0.05 vs. I/R with scramble group.

Previous studies reported that lncRNAs act as a competing endogenous RNA (ceRNA) to perform their functions [19,30]. Herein, we predicted that miR−10b−3p was one of the target miRNAs of lncRNA 148400 using the software RegRNA 2.0 (Figure 4A). Subsequently, dual luciferase reporter (DLR) assay showed that miR−10b−3p mimics suppressed the luciferase activity of lncRNA 148400-WT but not lncRNA148400-MUT (Figure 4B). FISH analysis showed that lncRNA 148400 and miR−10b−3p co-localized in the cell cytoplasm of both BUMPT cells and mouse kidney tissues under basic and I/R conditions (Figure 4C). Finally, the RT−qPCR analysis indicated that the expression of miR−10b−3p was reversed by the lncRNA 148400 siRNA. By contrast, this effect was enhanced by the overexpression of lncRNA 148400 under basic and I/R conditions (Figure 4D,E). Collectively, the data suggest that miR−10b−3p was a target of lncRNA 148400.

### 3.5. miR−10b−3p Protected against I/R-Induced Apoptosis

**Figure 5:**
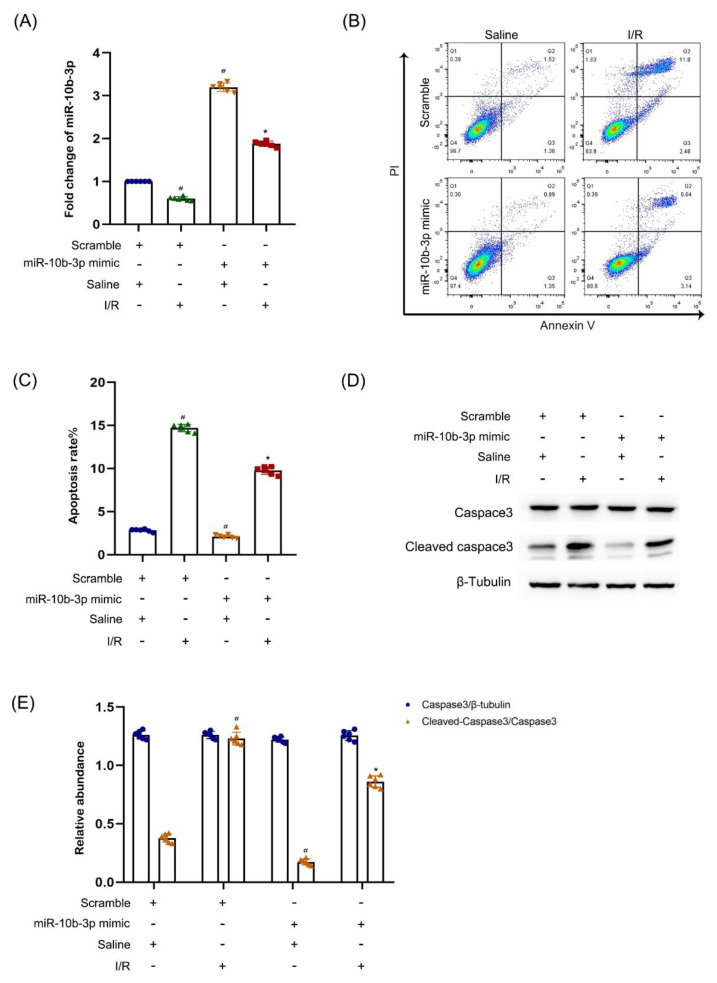
Overexpression of miRNA−10b−3p attenuated I/R-induced the apoptosis and the expression of cleaved−caspase 3 in BUMPT cells. BUMPT cells were transfected with 100 nM miRNA−10b−3pmimics or scramble, and then treated with or without I(2 h)/R(2 h) injury. (**A**) The RT−qPCR analysis of the expression of miRNA−10b−3p. (**B**) FCM analysis of apoptosis in BUMPT cells. (**C**) Apoptosis rate (%). (**D**) The immunoblot analysis of the expression of caspase 3 and cleaved-caspase 3. (**E**) The gray analysis between them. Data are expressed as mean ± SD (*n* = 6). # *p* < 0.05, I/R with scramble group vs. scramble group; * *p* < 0.05 vs. I/R with scramble group.

Although lncRNA148400 sponged the miR−10b−3p, the role of miR−10b−3p remains unclear. BUMPT cells were transfected with either scramble or miR−10b−3p mimics and subjected to I/R treatment. The miR−10b−3p level was markedly enhanced by mimics under basic and I/R conditions (Figure 5A). Functionally, miR−10b−3p markedly suppressed I/R-induced apoptosis in BUMPT cells (Figure 5B,C), which was further confirmed by immunoblot analysis of cleaved caspase−3 (Figure 5D,E). The data showed that miR−10b−3p has an anti-apoptotic role.

### 3.6. GRK4 Was a Target Gene of miR−10b−3p

**Figure 6:**
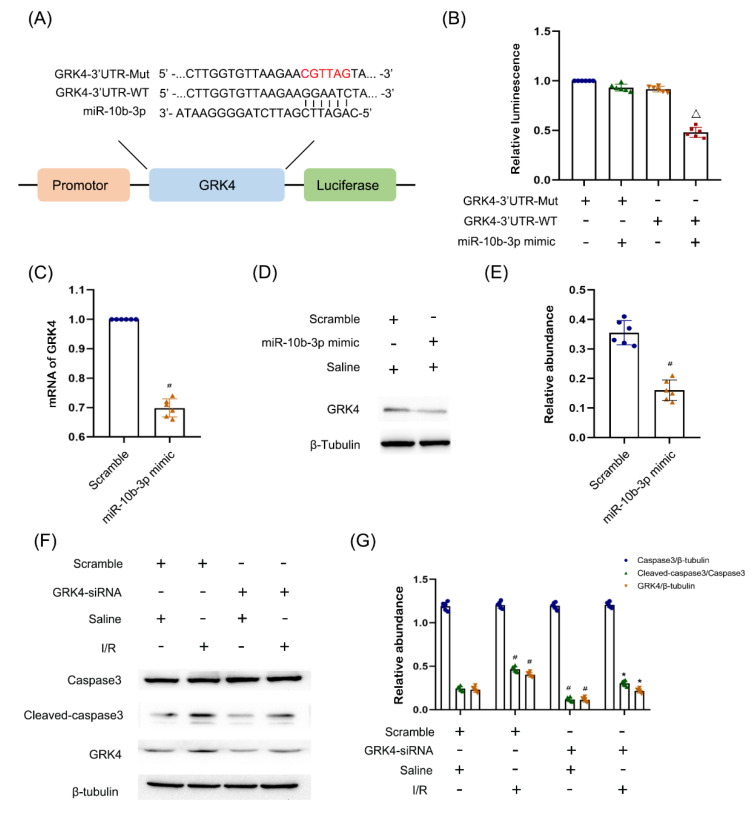
**GRK4 was a direct target gene of miRNA−10b−3p**. BUMPT cells were transfected with 100 nM miRNA−10b−3pmimics plus with or without GRK4−WT or Mut plasmid for 24 h. BUMPT cells were transfected with GRK4 siRNA, and then treated with I(2 h)/R(2 h) injury. (**A**) Representative miR−10b−3p complementary binding sites or mutated sequence in the 3′UTR of GRK4 mRNA. (**B**) Representative the luciferase activities. (**C**) RT−qPCR analysis of the expression of GRK4. (**D**) Immunoblot analysis of GRK4 andβ−tubulin. (**E**) The gray analysis between them. (**F**) Immunoblot analysis of Cleaved−caspase3, Caspase3, GRK4, and β-tubulin. (**G**) The gray analysis between them. Data are expressed as mean ± SD (*n* = 6). Δ*p* < 0.05 vs. co-transfection of miR−10b−3p mimic and GRK4-3′UTR-WT or GRK4-3′UTR-MUT groups, # *p* < 0.05 vs. saline with scramble group; * *p* < 0.05 vs. I/R with scramble group.

Although miR−10b−3p suppressed apoptosis, its underlying mechanism is unclear. Using the website mirdb.org, we predicted that GRK4 was one of the target genes of G protein-coupled receptor kinase (GRK4). Figure 6 shows the sequence of complementary and mutated sites of GRK4 and miR−10b−3p (Figure 6A,B). In addition, miR−10b−3p mimics significantly suppressed the mRNA and protein levels of GRK4 in BUMPT cells (Figure 6C–E). Finally, we evaluated the function of GRK4. The immunoblot results demonstrated that GRK4 siRNA significantly reduced the I/R-induced expression of cleaved caspase−3 (Figure 6F,G), which showed that GRK4 was an apoptosis inducer. These data indicated that miR−10b−3p targeted GRK4 to prevent apoptosis.

### 3.7. LncRNA 148400 siRNA Attenuated I/R-Induced BUMPT Cell Apoptosis, Which Was Reversed by miR−10b−3p Inhibitor

**Figure 7:**
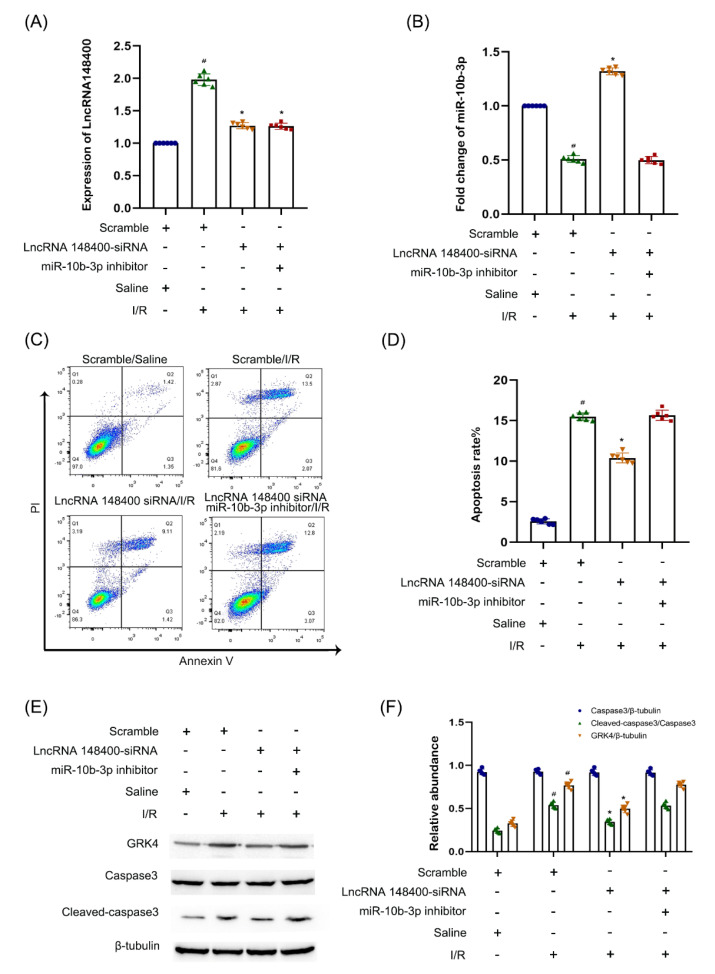
**Knock down of LncRNA148300 attenuated the I/R induced BUMPT cell apoptosis was reversed by anti−miR−10b−3p**. BUMPT cells were co-transfected with100 nM lncRNA148400 siRNA plus with or without anti−miR−10b−3p, and then treated with I(2 h)/R(2 h) injury. (**A**) RT−qPCR analysis the expression of LncRNA148400. (**B**) RT−qPCR analysis of the expression of miR−10b−3p. (**C**) FCM analysis of apoptosis in BUMPT cells. (**D**) Representative apoptosis rate (%). (**E**) Immunoblot analysis of the expression of Caspase 3, cleaved-caspase3, GRK4, and β−tubulin. (**F**) The gray analysis between them. Densitometric measurement of protein signals. Data are expressed as mean ± SD (*n* = 6). # *p* < 0.05 vs. saline with scramble group; * *p* < 0.05 vs. I/R with LncRNA148400 siRNA group.

The rescue experiment was used to confirm whether miR−10b−3p mediated the function of lncRNA 148300. The RT−qPCR analysis results showed that lncRNA 148400 siRNA markedly suppressed I/R-induced expression of lncRNA 148400, which was not affected by the miR−10b−3p inhibitor (Figure 7A). LncRNA 148400 siRNA reversed the I/R-induced downregulation of miR−10b−3p, which was prevented by the miR−10b−3p inhibitor (Figure 7B). FCM and immunoblot analysis showed that lncRNA 148400 siRNA attenuated the I/R−induced apoptosis as well as the increase in GRK4 and cleaved caspase−3 in BUMPT cells. However, this effect was reversed by the miR−10b−3p inhibitor (Figure 7C–F). The data provide strong evidence that miR−10b−3p was a key target of lncRNA 148400.

### 3.8. LncRNA 148400 siRNA Attenuates I/R-Induced AKI in Mice

**Figure 8:**
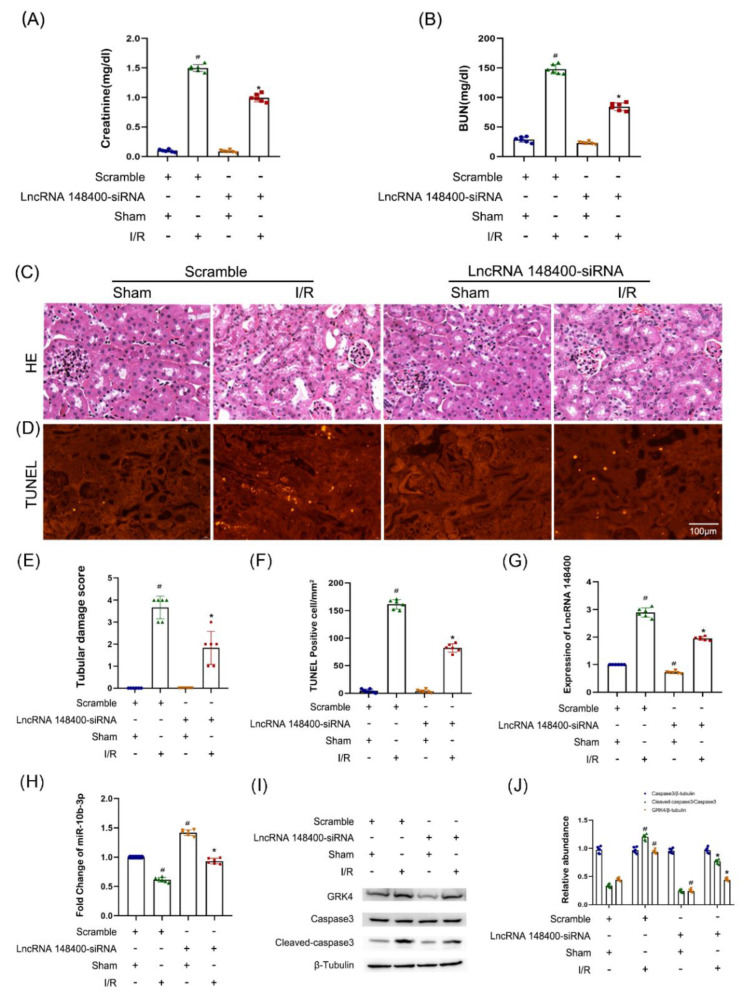
**LncRNA148400 siRNA ameliorates I/R**−**induced mice AKI.** The C57BL/6J mice were injected with lncRNA148400 siRNA via tail vein for 12 h, and then the bilateral renal pedicles were subjected to I(28 min)/R(48 h). (**A**) Creatinine. (**B**) BUN. (**C**) HE staining. (**D**) TUNEL. (**E**) Tubular damage scores. (**F**) TUNEL−positive cells. (**G**,**H**) RT−qPCR analysis of the expression of lncRNA148400 and miR−10b−3p. (**I**) Immunoblot analysis of caspase 3, cleaved−caspase3, GRK4, and β-tubulin. (**J**) The gray analysis between them. Data are expressed as mean ± SD (n = 6). # *p* < 0.05 vs. Sham with scramble group, * *p* < 0.05 vs. I/R with scramble group.

We further evaluated the function of lncRNA 148400 in mice with I/R-induced AKI. LncRNA 148400 siRNA or saline was injected via the tail vein for 12 h, and the mice were subjected to I/R (28 min and 48 h). LncRNA 148400 siRNA significantly reduced the I/R-induced increase in both serum Cr levels and BUN (Figure 8A,B). In line with these results, H&E and TUNEL staining showed that lncRNA 148400 siRNA significantly ameliorated the I/R-induced renal tubular damage and apoptosis, respectively (Figure 8C–F). The RT−qPCR analysis showed that lncRNA 148400 expression was silenced by lncRNA 148400 siRNA. By contrast, miR−10b−3p expression was reversed by lncRNA 148400 siRNA under sham and I/R treatment conditions (Figure 8G,H). Finally, lncRNA siRNA suppressed the I/R-induced increase in GRK4 and cleaved caspase−3 (Figure 8I,J). Taken together, these data suggest that the lncRNA 148400/miR−10b−3p/GRK4 axis mediated the progression of ischemic AKI.

## 4. Discussion

The role of lncRNAs in ischemic AKI remains largely unknown. Herein, we found that lncRNA 148400 promotes renal cell apoptosis in BUMP cells after ischemic injury. Furthermore, we found that lncRNA148400 acted as a ceRNA to suppress miR−10b−3p expression and increased GRK4 expression. Finally, the lncRNA 148400/miR−10b−3p/GRK4 axis mediated the progression of ischemic AKI.

Recent studies have reported that lncRNAs are involved in the regulation of renal cell apoptosis. Several lncRNAs, including H19, TUG1, TCONS_00016406, and PRNCR1, suppressed the I/R-induced renal cell apoptosis [15,16,17,18]. However, other lncRNAs, such as Meg3, Malat1, GAS5, NEAT1, and LOC105374325 mediated the renal cell apoptosis caused by ischemia [10,11,12,13,14]. The present study is the first to report that lncRNA 148400 mediated the renal cell apoptosis caused by ischemic injury. In particular, we found that lncRNA 148400 siRNA or overexpression attenuated or enhanced renal cell apoptosis during I/R treatment, respectively (Figure 2, Figure 3 and Figure 8). Taken together, these data suggest that lncRNA 148400 induces apoptosis during ischemic injury.

For most lncRNAs, ceRNAs are a key mechanism involved in their function. The prediction using RegRNA 2.0 software and DLR assay demonstrated that lncRNA 148400 directly binds to miR−10b−3p (Figure 4A,B), which was further confirmed by co-localization of lncRNA 148400 and miR−10b−3p (Figure 4C). Interestingly, RT−qPCR showed that the expression of miR−10b−3p was negatively regulated by lncRNA 148400 under basic and I/R treatment conditions (Figure 4D,E). The forementioned data confirmed that miR−10b−3p was a target of lncRNA 148400.

A recent study demonstrated that miR−10b−3p prevented I/R-induced brain cell apoptosis during cerebral injury [38]. In line with this, the present study showed that miR−10b−3p alleviated I/R-induced renal tubular cell apoptosis (Figure 5). Previous studies reported that both Krüppel-like factor 5 (KLF5) and FOXO3 were downstream factors of miR−10b−3p [38,39]. We found that GRK4, a member of the G protein-coupled receptor kinases, is a target of miR−10b−3p, based on the DLR analysis and regulation experiments of miR−10b−3p and GRK4 (Figure 6). A recent study reported that GRK4 promoted cardiomyocyte apoptosis [40]. Consistent with this, we also demonstrated that GRK4 knockdown reduced I/R-induced renal tubular cell apoptosis (Figure 6). Finally, the rescue experiment confirmed that miR−10b−3p/GRK4 was a key downstream factor of lncRNA 148400 (Figure 7), which was further verified by silencing of lncRNA 148400 in mice ischemic AKI (Figure 8).

In conclusion, we found a novel pathogenesis of ischemic AKI, i.e., the lncRNA 148400/miR-378a-3p/Rab10 axis promotes renal cell apoptosis to mediate the progression of ischemic AKI.

## Data Availability

All data generated or analyzed during this study are included in this published article.

## Supplementary Materials

The following supporting information can be downloaded at: https://www.mdpi.com/article/10.3390/cells11243986/s1, Figure S1: The lncRNA ENSMUST_147219 was induced by I/R in vivo.

## Abbreviations

RIRI	Renal ischemia−reperfusion injury;
BUMPT	Boston university mouse proximal tubule;
AKI	acute kidney injury:
LncRNA	long non−coding RNA;
I/R	ischemia/reperfusion;
FCM	flow cytometry;
BUN	blood urea nitrogen
FISH	Fluorescence in situ hybridization;
TUNEL	TdT−mediated dUTP nick end labeling
GRK4	G Protein−Coupled Receptor Kinase 4
