# Supplementary material for: LncRNA 148400 Promotes the Apoptosis of Renal Tubular Epithelial Cells in Ischemic AKI by Targeting the miR−10b−3p/GRK4 Axis
Source: Cells. 2022 Dec 9;11(24):3986. doi: 10.3390/cells11243986 (PMC9776552; doi:10.3390/cells11243986)
Supplement: Supplementary file 1 [file cells-11-03986-s001.zip › cells-1997800-supplementary.pdf]

LncRNA 148400 promotes the apoptosis of renal tubular epithelial cells in ischemic AKI by targeting the miR-10b-3p/GRK4 axis

Xingjin Li [1,2,3,†], Zhifen Wu [4,†], Jurong Yang [4,*] and Dongshan Zhang [1,2,3,*]

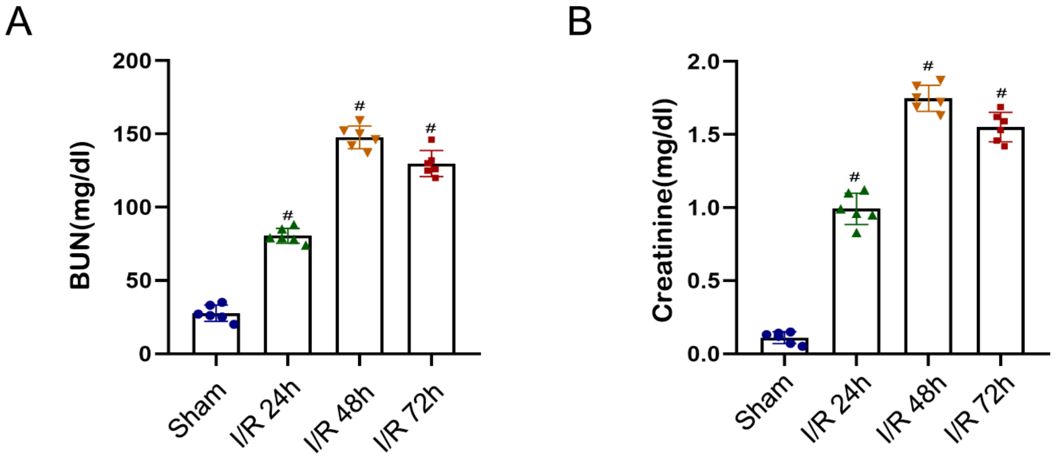

Supplementary Figure S1: The lncRNA ENSMUST_147219 was induced by I/R in vivo

C57BL/6J mice were subjected to I(28min)/R(24h,48h,72h) treatment. Blood samples were collected for the measurement of blood urea nitrogen (BUN) (A) and serum creatinine(B) concentrations at 24 h, 48 h and 72h. $^{\#}$ $p < 0.05$.
